# Supplementary material for: UBL3 modification influences protein sorting to small extracellular vesicles
Source: Nat Commun. 2018 Sep 26;9:3936. doi: 10.1038/s41467-018-06197-y (PMC6158211; doi:10.1038/s41467-018-06197-y)
Supplement: Supplementary file 1 — Supplementary Information [file 41467_2018_6197_MOESM1_ESM.pdf]

Supplementary Information

UBL3 modification influences protein sorting to small extracellular vesicles

Ageta et al.

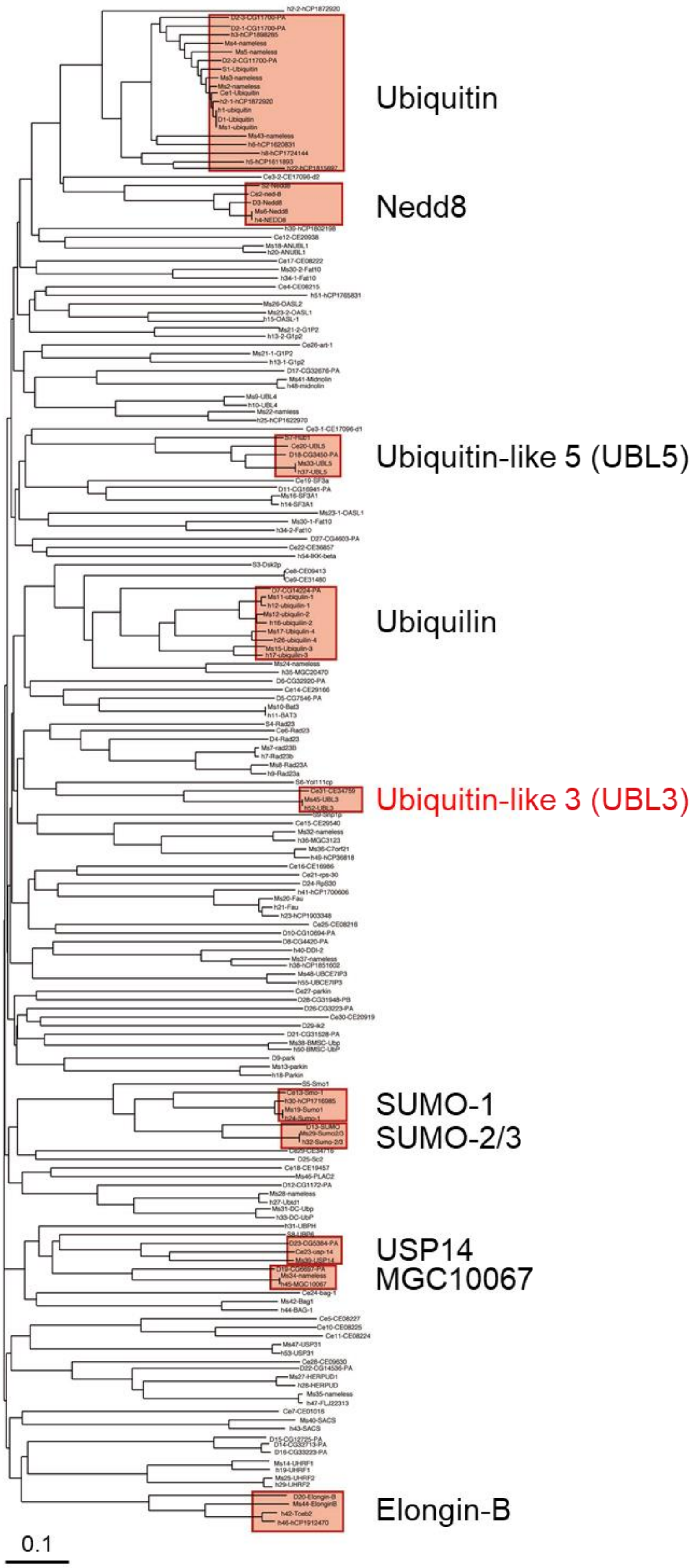
Ubiquitin
Nedd8
Ubiquitin-like 5 (UBL5)
Ubiquilin
Ubiquitin-like 3 (UBL3)
SUMO-1
SUMO-2/3
USP14
MGC10067
Elongin-B
0.1

Supplementary Figure 1

Phylogenetic tree analysis of ubiquitin-like (UBL) domain-containing proteins from various species. All the UBL amino acid sequences in humans, mice, flies, worms, and yeasts were analysed using CLUSTALW (http://www.ebi.ac.uk/clustalw) and categorised into clusters to create a phylogenetic tree. The average distance between any two terminal taxa in the tree was calculated as follows:

Average distance = 2 × (the sum of all branches) / (the number of UBL domains – 1)

= 2 × (41.44) / (181 – 1)

= 0.46.

Based on this calculation, we categorised UBL domain-containing proteins into subfamilies as follows. First, we studied the distances between each of the UBL domains, including the branch length of the internal nodes. If the distance was under 0.46, we included the two domains in the same group and defined the group as a subfamily of the UBL domain. We then defined a highly conserved subfamily of the UBL domain among humans, mice, and either worms, flies, or yeasts. Orange boxes indicate the highly conserved subfamily of the UBL domain, namely, Ubiquitin, Nedd8, Ubiquitin-like 5 (UBL5), Ubiquilin, Ubiquitin-like 3 (UBL3), SUMO-1, SUMO-2/3, Ubiquitin-specific protease 14 (USP14), MGC10067, and Elongin-B.

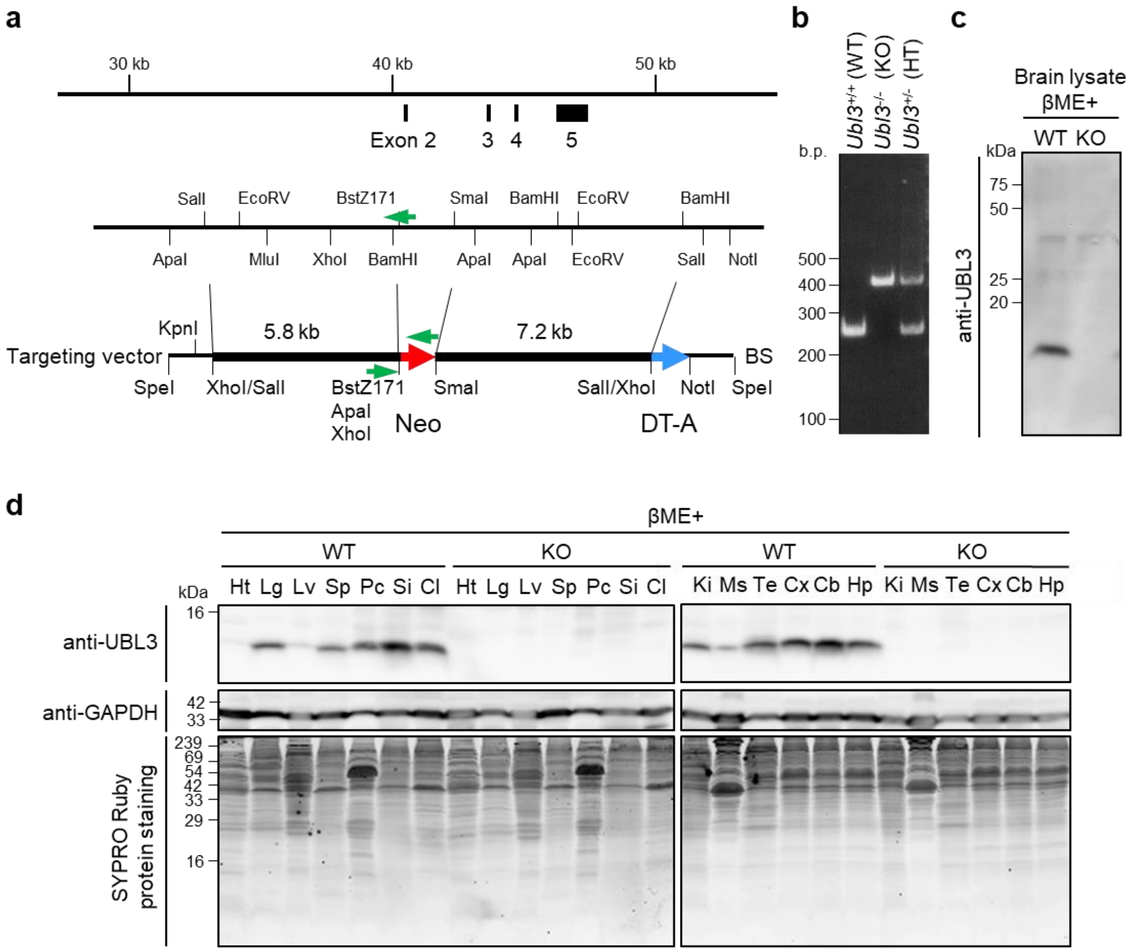

Supplementary Figure 2

Generation of *UBL3*-null mice. **a**, Schematic structure of the targeting vector to remove exon 2 of the UBL3 gene, which contains an initiation codon. Red arrow, neomycin-resistance (Neo) gene. Blue arrow, diphtheria toxin A (DT-A) gene. Green arrows, the positions of PCR primers for the genotyping analysis. BS, pBluescript. Targeted clones were confirmed by Southern blotting analysis of KpnI–HindIII-digested genomic DNA; probed with the 5' probe using a 750-bp fragment downstream to the KpnI site external to the 5' position of the targeting construct; and for NcoI-digested genomic DNA, probed using the 3' probe using an 800-bp fragment between the NdeI and the NcoI sites at the 3' end. **b**, Genotype analysis of the *UBL3* KO mice. **c**, Western blot of UBL3 in the brain from *Ubl3*$^{+/+}$ (wild-type; WT) and *Ubl3*$^{-/-}$ (knockout; KO) mice with anti-UBL3 antibodies. Twenty µg per lane. **d**, Tissue distribution of endogenous UBL3 protein was examined by western blotting analysis with UBL3 antiserum. GAPDH was used as a loading control. After the western blot analysis, the gels were stained with SYPRO Ruby. Ht, heart; Lg, lung; Lv, liver; Sp, spleen; Pc, pancreas; Si, small intestine; Cl, colon; Ki, kidney; Ms, skeletal muscle; Te, testis; Cx, cerebral cortex; Cb, cerebellum; Hp, hippocampus. Fifty µg per lane.

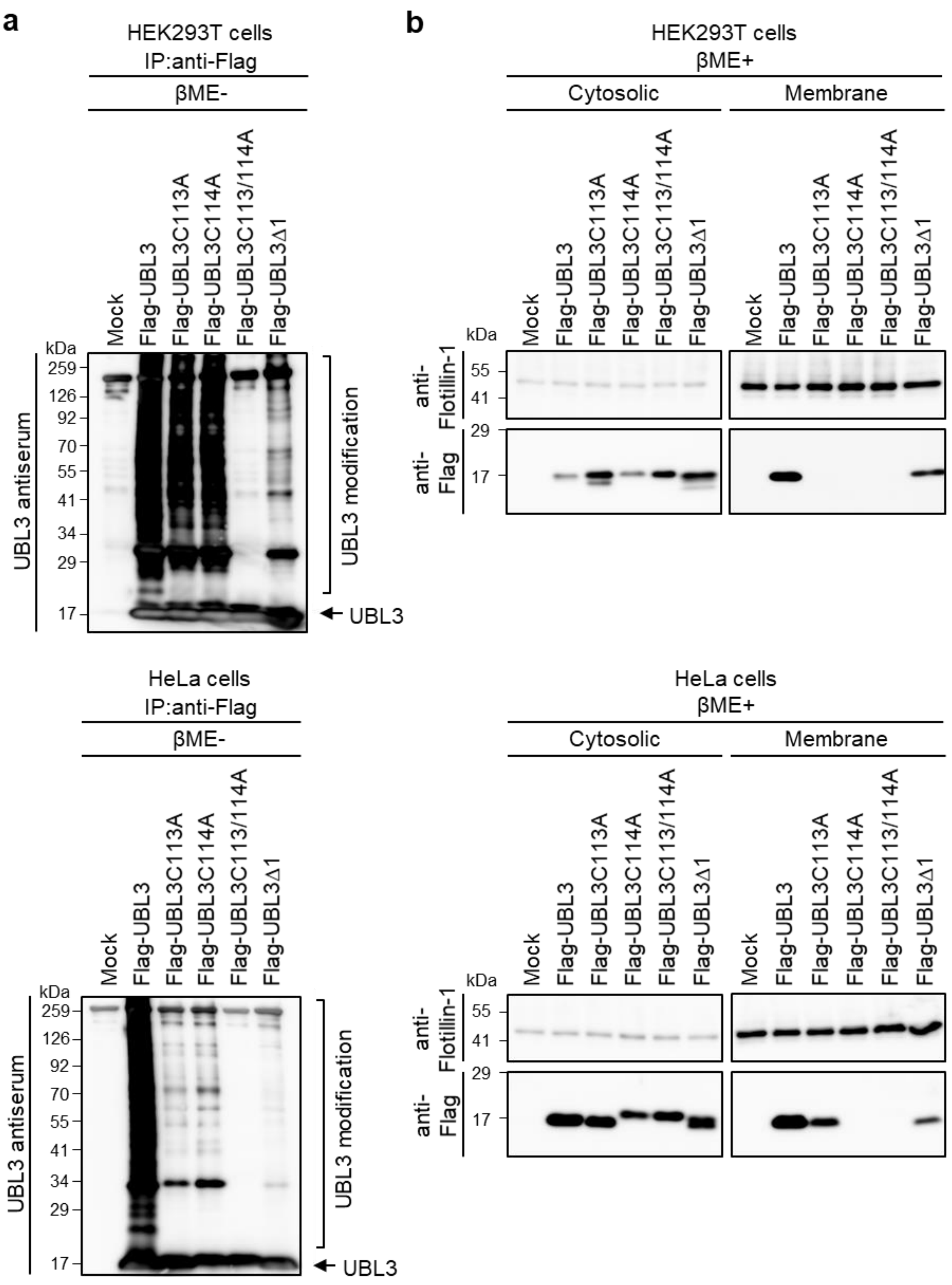

Supplementary Figure 3

Analysis of UBL3 modification in HEK293T and HeLa cells. a, Detection of UBL3 modification in HEK293T and HeLa cells. IP products were boiled without 2-mercaptoethanol (βME-) before loading. **b**, Subcellular localisation of UBL3 in HEK293T and HeLa cells transfected with Flag-UBL3 (wild-type and mutants). Ten μg per lane.

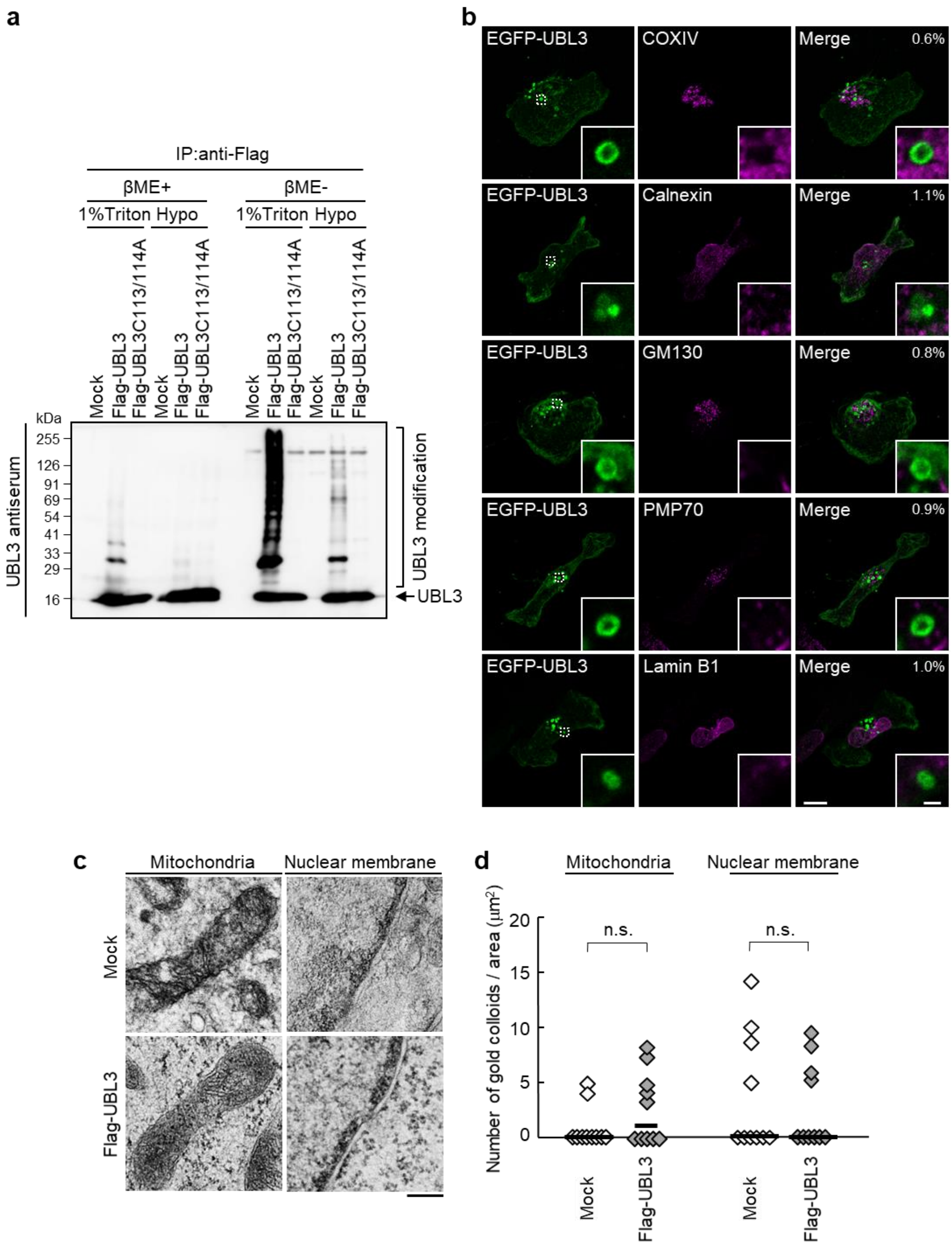

Supplementary Figure 4

Subcellular localisation of UBL3. **a**, PTM products by UBL3 are reduced in the cytoplasm in the cells. MDA-MB-231 cells transfected with mock, Flag-UBL3, and Flag-UBL3C113/114A vectors were lysed with 1% Triton or Hypotonic (Hypo) buffer and subjected to analysis of UBL3 modification. **b**, Representative images of MDA-MB-231 cells transfected with EGFP-UBL3 and co-stained with markers for mitochondria (COXIV), endoplasmic reticulum (Calnexin), Golgi (GM130), peroxisomes (PMP70), or nuclear membrane (Lamin B1). Values in merged images are shown as % of total UBL3. n = 5 x 5. Scale bars, 10 μm and 1 μm. **c**, Immune-EM images of Flag-UBL3 in mitochondria and nuclear membrane of MDA-MB-231 cells. **d**, Quantification of the numbers of gold colloids per area in c. n = 10. n.s., $p > 0.05$ by two-tailed Student's t-tests.

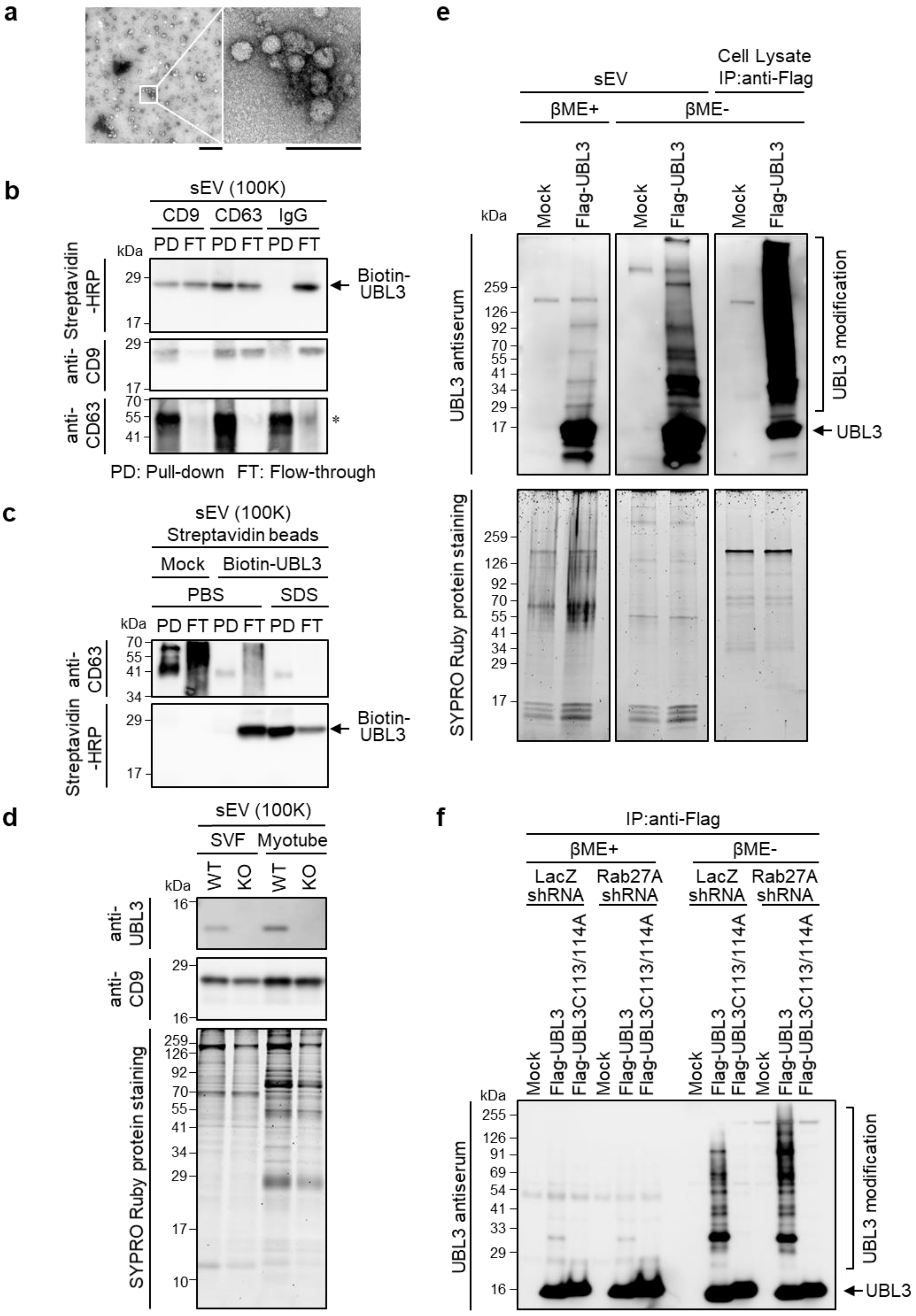
a
b
sEV (100K)
CD9 CD63 IgG
PD FT PD FT PD FT
kDa
Streptavidin -HRP
anti- CD9
anti- CD63
Biotin- UBL3
*
PD: Pull-down FT: Flow-through
c
sEV (100K)
Streptavidin beads
Mock Biotin-UBL3
PBS SDS
PD FT PD FT PD FT
anti- CD63
Streptavidin -HRP
Biotin- UBL3
d
sEV (100K)
SVF Myotube
WT KO WT KO
anti- UBL3
anti- CD9
SYPRO Ruby protein staining
e
sEV
Cell Lysate
IP:anti-Flag
βME+ βME-
Mock Flag-UBL3 Mock Flag-UBL3 Mock Flag-UBL3
UBL3 antiserum
UBL3 modification
UBL3
SYPRO Ruby protein staining
f
IP:anti-Flag
βME+ βME-
LacZ shRNA Rab27A shRNA LacZ shRNA Rab27A shRNA
Mock Flag-UBL3 Flag-UBL3C113/114A
UBL3 antiserum
UBL3 modification
UBL3

Supplementary Figure 5

Analysis of UBL3 in the sEVs. a, Electron microscopic analysis of the 100K pellet (sEVs). Right panel, magnified regions from the white box in the left panel. Scale bars, 500 nm and 200 nm. **b**, Immuno-isolation analysis. MDA-MB-231 cells transfected with UBL3 in a biotinylation tag vector (BioEase) were subjected to pull-down experiments using anti-CD9 and anti-CD63 antibodies. When the sEVs were immunoprecipitated either with anti-CD9 or anti-CD63 antibodies as described in the immuno-isolation methods of Kowal J et al.[1], the UBL3 signal was found in the pull-down (PD) fraction. On the other hand, UBL3 is only found in the flow-through (FT) fraction in the control IgG pull-down sample. Equal volumes of PD and FT fractions were loaded on gels for western blot analysis. *, nonspecific signal from the immunoglobulins' heavy chain (50 kDa) used for immuno-precipitation. **c**, The detection of UBL3 within sEVs. Under the PBS treatment, biotinylated UBL3 was identified in the FT fraction but not in the PD fraction. On the other hand, the majority of transfected biotinylated UBL3 was identified in the PD fraction with SDS treatment, indicating that UBL3 was packaged inside the sEVs. Equal volumes of the PD and FT fractions were loaded on gels for western blot analysis. **d**, Endogenous UBL3 protein was found in the sEVs. Western blotting analysis of the sEVs from the cell culture medium of the primary SVF and myotubes isolated from WT and *UBL3* KO mice. Before sample loading, the samples were boiled with βME. Ten percent of the purified sEVs from each condition were loaded and analysed. **e**, UBL3 modification in the sEVs. The sEVs were isolated from the culture medium of MDA-MB-231 cells transfected with Flag-tagged UBL3. The same amounts of sEVs (1 μg of protein) were blotted with UBL3 antiserum. IP products from cell lysates were loaded as positive controls for UBL3 modification. **f**, Effects of genetic inhibition of sEV release by Rab27a shRNA on the UBL3 modification. MDA-MB-231 cells were transfected either with LacZ shRNA or Rab27a shRNA. After 72 h, the effect of Rab27a shRNA on UBL3 modification was studied.

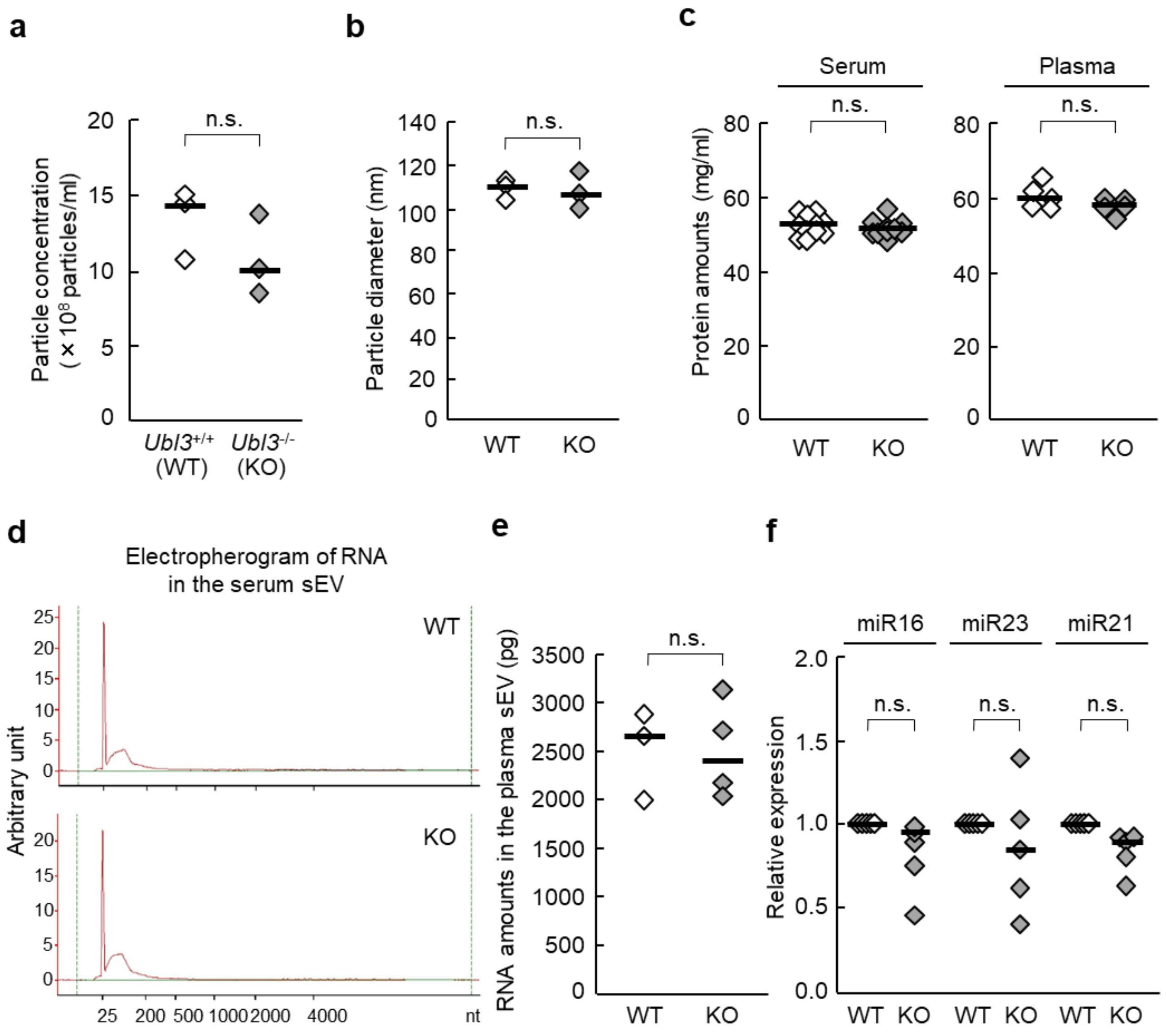

Supplementary Figure 6

Analyses of sEVs from serum and plasma from WT and *UBL3* KO mice. a, b, Measurements of sEV particles using NanoSight in serum sEVs from WT (n = 3) and UBL3 KO (n = 3) mice. **a**, Particle concentration. **b**, Particle diameter. **c**, Total protein concentration in serum and plasma from WT (white square; serum, n = 11; plasma, n = 5) and *UBL3* KO mice (grey square; serum, n = 10; plasma, n = 5). There is no significant difference between genotypes (n.s., $p > 0.05$ by Wilcoxon signed-rank test). **d**, Electropherogram data on total RNA in the serum sEVs. **e**, Total RNA amounts in the plasma sEVs were measured using a Bioanalyzer 2100. Each diamond-shaped spot indicates total RNA amounts in the plasma sEVs purified from WT (white square, n = 3) and *UBL3* KO mice (grey square, n = 4). There was no significant difference between genotypes (n.s., $p > 0.05$ by Mann-Whitney test). **f**, Real-time qPCR analysis of the miRNAs in the serum sEVs purified from WT and UBL3 KO mice. n = 5. n.s., $p > 0.05$ by Mann-Whitney test.

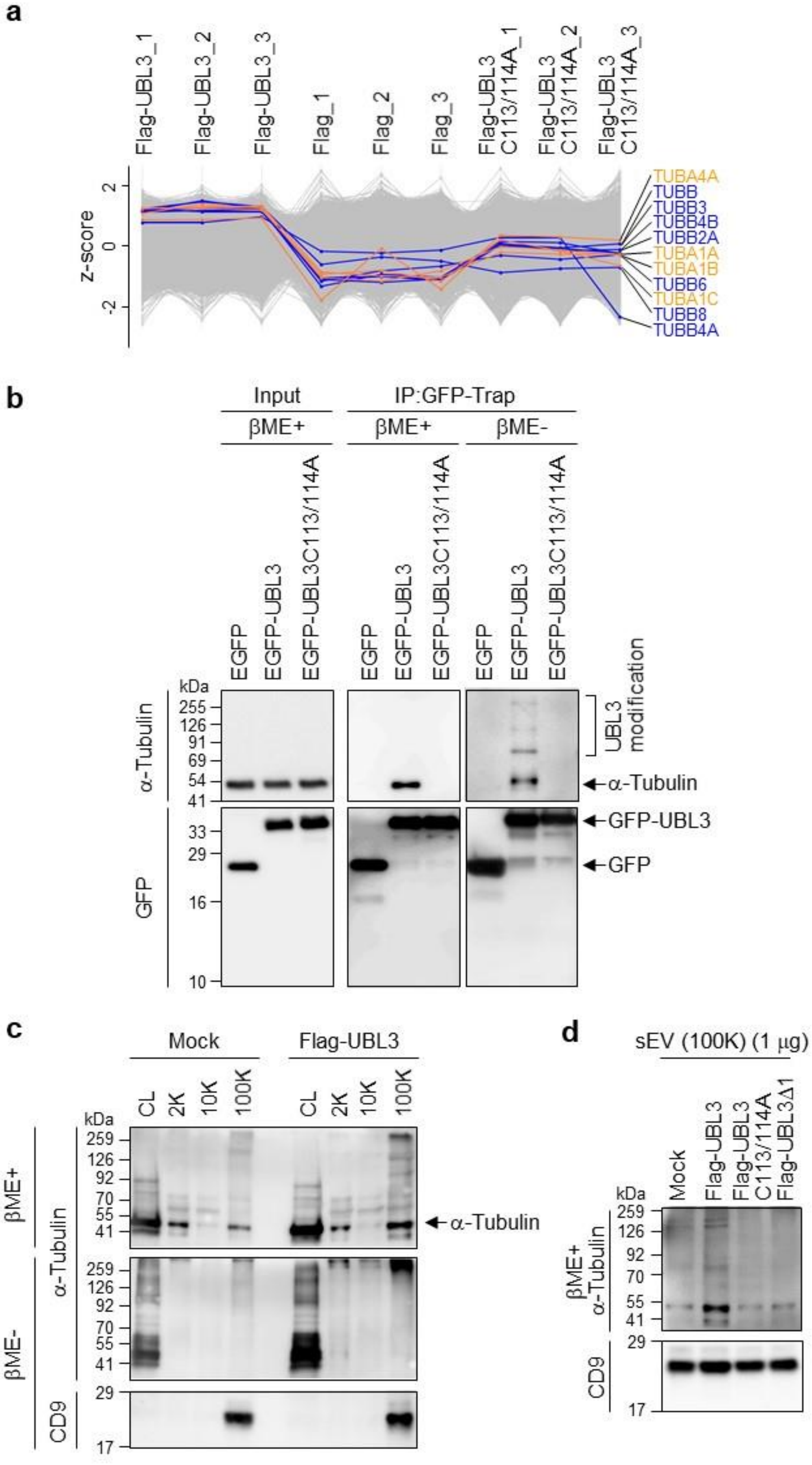

a
Flag-UBL3_1
Flag-UBL3_2
Flag-UBL3_3
Flag_1
Flag_2
Flag_3
Flag-UBL3 C113/114A_1
Flag-UBL3 C113/114A_2
Flag-UBL3 C113/114A_3
z-score
TUBA4A
TUBB
TUBB3
TUBB4B
TUBB2A
TUBA1A
TUBA1B
TUBB6
TUBA1C
TUBB8
TUBB4A
b
Input
IP:GFP-Trap
βME+
βME+
βME-
EGFP
EGFP-UBL3
EGFP-UBL3C113/114A
kDa
α-Tubulin
GFP
UBL3 modification
←α-Tubulin
←GFP-UBL3
←GFP
c
Mock
Flag-UBL3
CL
2K
10K
100K
βME+
βME-
α-Tubulin
CD9
←α-Tubulin
d
sEV (100K) (1 μg)
Mock
Flag-UBL3
Flag-UBL3 C113/114A
Flag-UBL3Δ1
βME+ α-Tubulin
CD9

Supplementary Figure 7

Posttranslational modification and sorting of tubulin to sEVs by UBL3.

a, The profiles of tubulin proteins in three conditions (3xFlag-UBL3, or 3xFlag empty vector, or 3xFlag-UBL3C113/114A) with triple biological replicates on the proteomics analysis. TUBA1A, 1B, 1C, and 4A are highlighted in orange. TUBB, B2A, B3, B4A, B4B, B6, and B8 are highlighted in blue. **b**, UBL3-dependent posttranslational modification by GFP-trap analyses. MDA-MB-231 cells were transfected with GFP-tagged UBL3 or UBL3C113/114A. Cell lysates were subjected to IP by GFP-trap, and the resulting immunoprecipitates were subjected to western blot analyses with anti-alpha-tubulin or anti-GFP antibodies. 2x sample buffer (without βME) was added to the beads, and the beads were boiled for 3 min (βME-). A portion of the samples was treated with 2-mercaptoethanol (βME+). **c**, The cell lysate (CL) and pellets (2K = 2,000 x *g*; 10K = 10,000 x *g*; 100K = 100,000 x *g*) from the conditioned medium of MDA-MB-231 cells transfected with mock or 3xFlag-UBL3 vectors were blotted with anti-tubulin antibodies. **d,** sEVs from the conditioned medium of MDA-MB-231 cells transfected with mock, 3xFlag-UBL3, 3xFlag-UBL3C113/114A or 3xFlag-UBL3Δ1 vectors were blotted with anti-tubulin antibodies. b, 1 μg per lane.

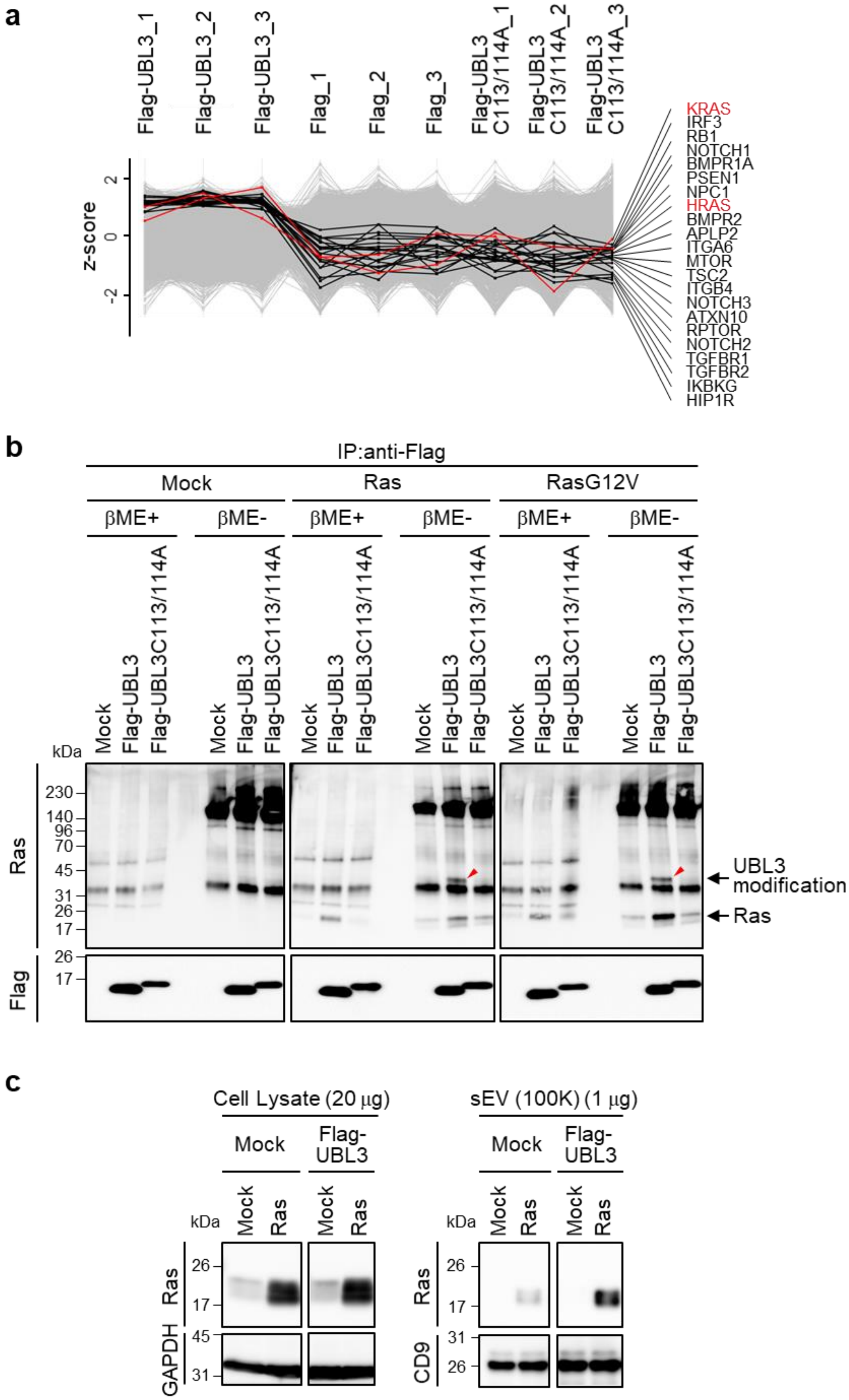
a
Flag-UBL3_1
Flag-UBL3_2
Flag-UBL3_3
Flag_1
Flag_2
Flag_3
Flag-UBL3 C113/114A_1
Flag-UBL3 C113/114A_2
Flag-UBL3 C113/114A_3
z-score
2
0
-2
KRAS
IRF3
RB1
NOTCH1
BMPR1A
PSEN1
NPC1
HRAS
BMPR2
APLP2
ITGA6
MTOR
TSC2
ITGB4
NOTCH3
ATXN10
RPTOR
NOTCH2
TGFBR1
TGFBR2
IKBKG
HIP1R
b
IP:anti-Flag
Mock
Ras
RasG12V
βME+
βME-
Mock
Flag-UBL3
Flag-UBL3C113/114A
kDa
230
140
96
70
45
31
26
17
Ras
Flag
UBL3 modification
Ras
c
Cell Lysate (20 μg)
sEV (100K) (1 μg)
Mock
Flag-UBL3
Mock
Ras
kDa
26
17
45
31
Ras
GAPDH
CD9

Supplementary Figure 8

Posttranslational modification and sorting of Ras proteins to sEVs by UBL3.

a, The profiles of disease-related proteins identified as UBL3-interacting proteins in three conditions (3xFlag-UBL3, 3xFlag empty vector, or 3xFlag-UBL3C113/114A) with triple biological replicates on the proteomics analysis. Disease-related molecules are highlighted in black. HRAS and KRAS are highlighted in red. **b**, MDA-MB-231 cells were transfected with wild-type Ras or oncogenic RasG12V, and either with 3xFlag-tagged UBL3 or UBL3C113/114A. Cell lysates were subjected to IP by anti-Flag antibody, and the resulting immunoprecipitates were subjected to western blot analyses with anti-Ras antibodies. 2x sample buffer (without βME) was added to the beads, and the beads were boiled for 3 min (βME-). A portion of the samples was treated with 2-mercaptoethanol (βME+). Red arrowheads, UBL3-modified Ras or RasG12V. **c**, The cell lysate and sEV from the conditioned medium of MDA-MB-231 cells transfected with mock or wild-type Ras and either with mock or 3xFlag-UBL3 vectors were blotted with anti-Ras antibodies.

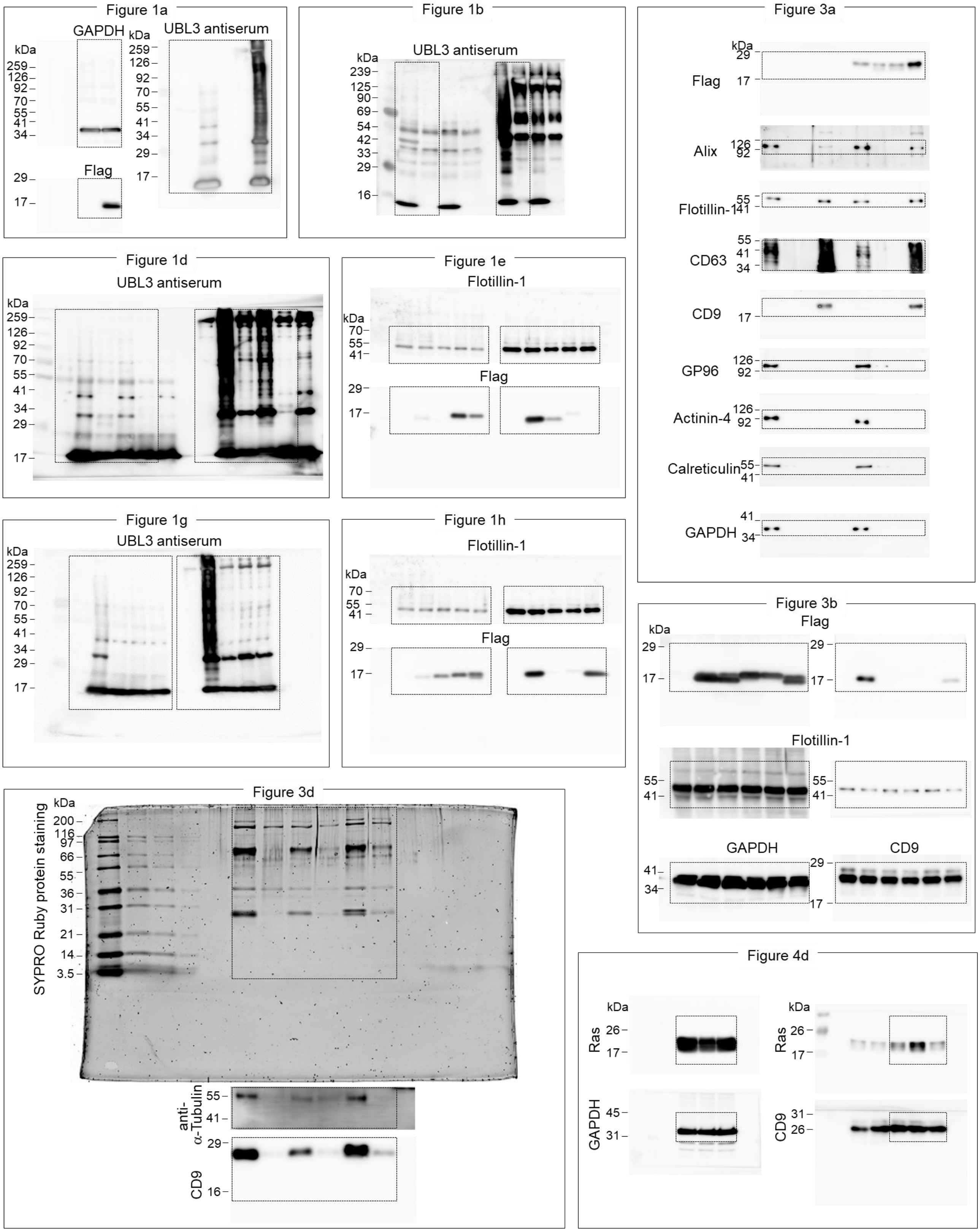

Supplementary Figure 9. Representative entire images of immunoblot

Boxed areas were cropped for designated figures.

Supplementary References

1 Kowal, J. *et al.* Proteomic comparison defines novel markers to characterize heterogeneous populations of extracellular vesicle subtypes. *Proc. Natl Acad. Sci. USA* **113**, E968-977 (2016).
