# Supplementary material for: UBL3 modification influences protein sorting to small extracellular vesicles
Source: Nat Commun. 2018 Sep 26;9:3936. doi: 10.1038/s41467-018-06197-y (PMC6158211; doi:10.1038/s41467-018-06197-y)
Supplement: Supplementary file 3 — Description of Additional Supplementary Files [file 41467_2018_6197_MOESM3_ESM.docx]

File Name: Supplementary Dataset

Description: List of all proteins identified by LFQ based proteomics analyses.
